# Clinical outcomes of liver transplantation in Caroli syndrome: a retrospective analysis

**DOI:** 10.1186/s13023-025-03943-6

**Published:** 2025-10-02

**Authors:** Fei Hou, Hao-Feng Xiong, Ying Liu, Shuang Zhao, Yi-Zhi Zhang, Qian Kang, Jing-Yi Liu, Lin Wei, Wei Qu, Zhi-Gui Zeng, Zhi-Jun Zhu, Li-Ying Sun

**Affiliations:** 1 Department of Critical Liver Diseases, National Clinical Research Center for Digestive Diseases, Beijing Friendship Hospital, Capital Medical University, 101 Luyuan East Road, Tongzhou District, Beijing, 101100 China; 2 Liver Transplantation Center, National Clinical Research Center for Digestive Diseases, Beijing Friendship Hospital, Capital Medical University, Beijing, China; 3 Laboratory for Clinical Medicine, Capital Medical University, Beijing, China; 4 Beijing Key Laboratory of Tolerance Induction and Organ Protection in Transplantation, Beijing, China

**Keywords:** Caroli syndrome, Liver transplantation, Living donor liver transplantation, Clinical outcomes, Long-term prognosis, Survival rate, Postoperative complications

## Abstract

**Objective:**

To evaluate the clinical outcomes of liver transplantation (LT) in patients with Caroli syndrome.

**Methods:**

We retrospectively analyzed clinical data from 20 patients diagnosed with Caroli syndrome who underwent LT at Beijing Friendship Hospital, Capital Medical University, between April 2014 and May 2024. Data included baseline characteristics, surgical parameters, complications, and survival. Follow-up concluded in February 2025.

**Results:**

These 20 cases accounted for 1.23% (20/1623) of all LTs during the study period. Thirteen patients received living donor liver transplants (LDLT), and seven received deceased donor liver transplants (DDLT). The cohort included 15 males and 5 females, aged 3–56 years (median 23.3). Common preoperative conditions included cirrhosis (*n* = 18), portal hypertension (*n* = 17), polycystic kidney disease (*n* = 16), and splenomegaly (*n* = 17). The median APACHE II score was 14; the predicted mortality averaged 26.0%. The median interval from the first major complication (e.g., recurrent cholangitis or decompensation) to LT was 12 months (IQR 4.5–21). No patients underwent cross-match or auxiliary LT; three had concurrent partial splenectomy. Postoperative complications included abdominal hemorrhage (*n* = 3) and seizures (*n* = 1). Chronic renal failure occurred in six patients during follow-up, and four experienced acute rejection (three recurrent). Compared with adults, pediatric patients had a higher proportion of females (*p* = 0.001), while chronic renal failure was more common in adults preoperatively (*p* = 0.010). Four patients (20%) died: one from heart failure (day 8), one from sudden death (6 months), one from pulmonary infection (1 year), and one from unknown causes (5.2 years). Median follow-up was 74.6 months (range 7-125). One-, three-, and five-year survival rates were 90%, 85%, and 78.5%, respectively.

**Conclusion:**

Liver transplantation significantly improves the survival prognosis for patients with Caroli syndrome, but attention to kidney function preservation and individualized immunosuppressive management is critical for optimal outcomes.

Caroli syndrome is a congenital disorder inherited in an autosomal recessive pattern, characterized by multifocal segmental dilation of the intrahepatic bile ducts, often accompanied by congenital hepatic fibrosis (CHF) and varying degrees of renal cystic diseases [1]. The treatment for Caroli syndrome is primarily supportive; however, patients who experience recurrent biliary infections, particularly those with complications related to portal hypertension, may require liver transplantation. There are limited reports on the clinical outcomes and long-term prognosis of liver transplantation for Caroli syndrome [2, 3]. This study uses a retrospective case analysis to summarize the clinical features and outcomes of 20 patients with Caroli syndrome who underwent liver transplantation, and to conduct follow-up evaluations to assess the efficacy of liver transplantation in managing this condition. To the best of our knowledge, this is the largest single-center series from mainland China, reflecting unique patient characteristics, surgical practices, and post-transplant outcomes in this population.

## Subjects

A search was conducted to identify patients who underwent liver transplantation at Beijing Friendship Hospital, Capital Medical University, between April 2014 and May 2024, and were discharged with a diagnosis of Caroli syndrome. The diagnostic criteria were as follows:

1. The diagnosis of Caroli disease was based on imaging findings (such as CT, MRI, or ultrasound), which revealed multiple cystic or cylindrical low-density lesions in the liver that communicate with the intrahepatic bile ducts, displaying the “tadpole sign” or “hanging sign,” while the common bile duct remains normal.
2. The diagnosis of Caroli syndrome was made when Caroli disease was complicated by portal hypertension, as evidenced by esophageal and/or gastric varices, portal hypertensive bleeding, and/or imaging studies showing portosystemic shunting [3].

## Methods

(1) Data Collection: The following data were collected from the patients: demographic information, laboratory test results (including liver and renal function, complete blood count, coagulation tests, and urinalysis), preoperative comorbidities (such as cirrhosis, portal hypertension, gastrointestinal bleeding, renal injury, ascites, hepatopulmonary syndrome, pulmonary hypertension, cholangitis, hypoalbuminemia, splenomegaly, etc.), surgical details (including organ source, whether a cross-match transplant was performed, whether splenectomy was done, whether auxiliary liver transplantation was used, graft weight, graft-to-recipient weight ratio, ischemia time, etc.), liver disease-related scores (such as Child-Pugh-Turcotte score, MELD score, PELD score, SOFA score, etc.), and postoperative complications (including respiratory failure, heart failure, chronic renal failure, rejection, and vascular complications).

Additionally, the patients were divided into two groups: pediatric (≤ 18 years) and adult (> 18 years). Differences between the two groups in terms of preoperative and postoperative comorbidities, as well as follow-up parameters, were compared. All patients were followed up postoperatively, with the follow-up period concluding in February 2025.

(2) Statistical Analysis: All statistical analyses were conducted using SPSS version 25.0 software. For normally distributed quantitative variables, data are presented as mean ± standard deviation (M ± SD), and group differences were assessed using the t-test. For non-normally distributed variables, data are presented as median (first quartile, third quartile) or Q2 (Q1, Q3), with group differences evaluated using the Mann-Whitney U test. Categorical variables are expressed as frequencies or percentages, with differences in proportions between groups compared using the chi-square test or Fisher’s exact test. A significance level of α = 0.05 was set, and *p* < 0.05 was considered statistically significant.

## Results

### General information

Between April 2014 and May 2024, a total of 20 patients diagnosed with Caroli syndrome underwent liver transplantation at Beijing Friendship Hospital, Capital Medical University, representing 1.23% (20/1623) of all liver transplant cases during this period. The ages of the patients at the time of surgery ranged from 3 to 56 years, with a median age of 8 years (6 years, 20.5 years). Of these, 13 patients (65%) received living-donor liver transplants (LDLT), and 7 patients (35%) received deceased-donor liver transplants (DDLT). The majority of deceased donor grafts(6 cases) were obtained from donors who suffered brain death due to traumatic brain injury or intracranial hemorrhage. The cohort consisted of 15 male patients and 5 female patients, with 15 patients (75%) being children and 5 patients (25%) being adults.

### Preoperative major comorbidities

Preoperative comorbidities included cirrhosis(18 patients), portal hypertension (17), splenomegaly (17), polycystic kidney disease (16), hypoalbuminemia (14), ascites (13), gastrointestinal bleeding (8), hepatomegaly (9), renal injury (2), pulmonary hypertension (3), hepatopulmonary syndrome (1), congenital liver fibrosis (2), and cholangitis (1). In our cohort of 20 patients, 18 underwent liver transplantation primarily due to decompensated cirrhosis and complications of portal hypertension (e.g., variceal bleeding, hypersplenism, refractory ascites). The remaining 2 patients did not have cirrhosis but experienced recurrent episodes of cholangitis that were refractory to percutaneous transhepatic cholangial drainage (PTCD) and antibiotic therapy. Both patients were pediatric cases with recurrent episodes of acute cholangitis and progressive disease despite supportive measures, which ultimately led to transplantation. Given the poor quality of life and failure of conservative management, liver transplantation was considered the definitive treatment. In our cohort, the median interval between the onset of the first severe complication (such as recurrent cholangitis or signs of liver decompensation) and liver transplantation was 12 months (IQR: 4.5–21 months).

When comparing adult and pediatric patients, females were more prevalent in the pediatric group, while chronic renal failure was more commonly observed in adults prior to surgery. No significant differences were found in other preoperative comorbidities. A detailed comparison of the general characteristics and preoperative major comorbidities of pediatric and adult patients is provided in Table 1.

**Table 1:** Comparison of general characteristics and preoperative major comorbidities between adult and pediatric patients with Caroli syndrome

	Adults (5 cases)	Children (15 cases)	Chi-square	*p*
Gender (F/M)	4/1	1/14	10.756	0.001^#^
Age (years)	37.6 ± 11.7	7.0 ± 3.3		
Prognosis			0.741	0.389
Cured	5	13		
Death	0	2		
Preoperative biliary infection	0	2	0.741	0.389
Hypersplenism	5	12	1.176	0.278
Hypoproteinemia	5	9	2.857	0.091
Liver cirrhosis	5	13	0.741	0.389
Portal hypertension	5	12	1.176	0.278
Gastrointestinal bleeding	1	7	1.111	0.292
Preoperative chronic kidney injury	2	1	6.667	0.010^#^
Ascites	3	10	0.073	0.787
Hepatopulmonary syndrome	2	1	4.157	0.125
Polycystic kidney	5	10	2.222	0.136
Pulmonary hypertension	2	1	3.268	0.071

Note: Compared to pediatric patients, adults had a higher proportion of females and were more prone to preoperative chronic kidney injury. No other preoperative comorbidities showed statistically significant differences

### Intraoperative conditions

The APACHE II score during surgery ranged from 4 to 27, with a mean of 14( 10.5,18.5), and the predicted mortality ranged from 5.1 to 72.5%, with a mean of 26.02% ( 20.92%, 44.53% ). The SOFA score ranged from 0 to 13, with a median of 4 (interquartile range 2.5 to 5.0), and the CLIF-SOFA score ranged from 0 to 7, with a median of 2 (interquartile range 0.5 to 4.5). The PELD score (for children under 12) ranged from 0 to 28, with a median of 11 (interquartile range 6 to 17.5), and the MELD score ranged from 9 to 24, with a mean of 16.7 ± 7.3. The graft weight ranged from 224 to 1327 g, with a mean of 325.5 g ( 268 g, 665 g), and the graft-to-recipient weight ratio (GRWR) ranged from 0.76 to 4.90%, with a mean of 1.7 ± 1.2%. The warm ischemia time ranged from 2 to 8 min, with a mean of 3 min ( 2 min, 5 min), and the cold ischemia time ranged from 30 to 657 min, with a mean of 120.5 min ( 88 min, 373.5 min). None of the patients received a cross-match transplant or auxiliary liver transplantation. Three patients underwent simultaneous partial splenectomy. There were no significant differences between adult and pediatric patients regarding donor source, graft type, surgical approach, and other intraoperative factors, as detailed in Table 2. In our cohort of 20 patients, 5 patients underwent hepatic-jejunostomy, and 15 patients underwent duct-to-duct anastomosis during liver transplantation. The choice of biliary anastomosis technique was determined based on intraoperative findings and the condition of the biliary tree.

**Table 2:** Comparison of surgical-related and postoperative major complications between adult and pediatric patients with Caroli syndrome

Category	Adults (5 cases)	Children (15 cases)	T	*p*
Organ Source			1.832	0.176
- LDLT	2	11		
- DDLT	3	4		
Splenectomy			0.131	0.718
- No splenectomy	4	13		
- Partial splenectomy	1	2		
Type of Living Graft			3.746	0.587
- Left lobe with MHV	1	5		
- Left lobe without MHV	0	1		
- Right lobe without MHV	1	1		
- Left lateral segment	0	3		
- Reduced-size left lateral	0	1		
Postoperative hypertension	0	1	0.741	0.389
Postoperative AKI	3	5	1.111	0.292
Postoperative acute rejection	1	3	0	1
Postoperative chronic renal failure	2	3	0.8	0.371
Respiratory failure	0	1	0.351	0.554
Heart failure	1	1	0.741	0.389
Portal vein stenosis	0	1	0.351	0.554
Hepatic artery stenosis	0	1	0.351	0.554
Intra-abdominal hemorrhage	2	1	3.268	0.071
Seizure	1	0	3.158	0.076
Cerebral infarction	1	0	3.158	0.076

Note: LDLT = Living donor liver transplantation; DDLT = Deceased donor liver transplantation; MHV = Middle hepatic vein; AKI = Acute kidney injury

There were no statistically significant differences between adult and pediatric patients in terms of donor source, graft type, surgical approach, or early postoperative complications

### Early postoperative complications

Early postoperative complications included hypertension in 1 patient (pediatric group), acute kidney injury in 8 patients (3 adults, 5 children), acute rejection in 1 patient (pediatric group), respiratory failure in 1 patient (pediatric group), abdominal hemorrhage in 3 patients (2 adults, 1 child), and seizures and cerebral infarction in 1 patient each (both in the adult group). One patient died during the initial hospitalization, resulting in a 5% mortality rate. The patient passed away on postoperative day 8 due to heart failure. No significant differences were observed in early postoperative complications between adult and pediatric patients, as detailed in Table 2.

### Long-term postoperative complications and follow-up

During the follow-up period of over one year post-surgery, the following long-term complications were observed: one case of portal vein stenosis (1 year post-surgery, pediatric group), one case of hepatic artery stenosis (1 year post-surgery, pediatric group), and one case of bowel obstruction (5 years post-surgery, pediatric group). Three patients experienced episodes of rejection, with the first acute rejection occurring at 1 year, 3.2 years, and 5.2 years post-surgery, all of which improved with treatment. However, the first two patients experienced a second acute rejection episode at 5 and 6 years post-surgery, respectively, both of which also responded to treatment. Chronic renal failure developed in 6 patients, with the onset ranging from 12.1 to 46.7 months post-surgery (detailed in Table 2). In our cohort, only one patient underwent both liver and kidney transplantation; however, the procedures were performed sequentially rather than simultaneously. This patient received a liver transplant in 2019 and subsequently underwent kidney transplantation six years later due to progressive renal dysfunction. None of the patients in our study were on dialysis prior to liver transplantation.

### Follow-up

In follow-up evaluations of patients monitored for more than 6 months, there were no significant differences in laboratory test results between adult and pediatric patients (as detailed in Table 3). Relevant data for patients with chronic renal failure are provided in Table 4. Of the total cohort, 4 patients (20%) died during the follow-up period. In the LDLT group, 3 patients died, while 1 patient in the DDLT group died, with no statistically significant difference in mortality rates between the two groups. The causes of death included heart failure on postoperative day 8, unexplained sudden death at 6 months post-surgery, respiratory failure due to pulmonary infection at 1 year post-surgery, and death from an unknown cause at 5.2 years post-surgery. Among the 16 surviving patients, the median follow-up time was 74.6 months (ranging from 7 to 125 months). The 1-year, 3-year, and 5-year survival rates for the entire cohort were 90%, 85%, and 78.5%, respectively.

**Table 3:** Comparison of major laboratory tests during post-transplant follow-up between pediatric and adult patients with Caroli syndrome

Parameter	Postoperative Last Follow-Up		T	*p*	Cohen’s d
	Children	Adults			
WBC (×10^9/L)	6.7 ± 4.0	6.9 ± 3.1	-0.127	0.900	0.067
Hgb (g/L)	130.7 ± 20.1	112.0 ± 20.5	1.76	0.097	0.926
PLT (×10^9/L)	199.8 ± 51.6	173.0 ± 77.6	0.862	0.402	0.453
PT (s)	13.8 ± 4.2	11.9 ± 2.2	0.922	0.370	0.485
PTA (%)	76.7 ± 19.2	90.0 ± 21.2	-1.258	0.226	0.662
INR	1.26 ± 0.38	1.11 ± 0.21	0.8	0.435	0.421
ALT (IU/L)	24.2 ± 15.3	12.4 ± 5.8	1.659	0.117	0.873
AST (IU/L)	30.2 ± 8.5	17.6 ± 6.0	3.029	0.008	1.594
TBIL (µmol/L)	12.7 ± 6.4	10.3 ± 2.7	0.798	0.436	0.420
DBIL (µmol/L)	4.1 ± 1.3	3.8 ± 0.5	0.521	0.609	0.274
Alb (g/L)	42.6 ± 2.4	40.0 ± 2.5	2.007	0.062	1.056
ALP (IU/L)	309.6 ± 288.7	177.0 ± 122.6	0.979	0.342	0.515
GGT (IU/L)	41.5 ± 56.3	40.6 ± 31.3	0.0335	0.973	0.018
BUN (µmol/L)	8.1 ± 5.7	13.2 ± 9.5	-1.403	0.180	0.738
Cr (µmol/L)	98.4 ± 87.1	221.7 ± 150.2	-2.199	0.043	1.157

**Table 4:** Summary of clinical data for patients with combined renal impairment

No.	Gender	Age (Surgery)	Surgery Date	Polycystic Kidney	Liver Donor Type	Splenectomy	Preoperative Renal Injury	Preoperative Peak Cr (µmol/L)	Postoperative Renal Injury	Postoperative Peak Cr (µmol/L)	Follow-Up Outcome
1	Male	11	2015-10-03	Yes	Deceased donor	No splenectomy	Yes	105.9	Yes	154	Liver and kidney function normalized
2	Male	8	2017-08-11	Yes	Left lobe	Partial splenectomy	No	58.2	Yes	126	Death (5 years post-op, November 2022, cause unknown)
3	Female	56	2018-07-01	Yes	Deceased donor	No splenectomy	Yes	130	Yes	130	Liver and kidney function normalized
4	Male	8	2018-12-25	Yes	Left lateral segment	No splenectomy	No	99.2	Yes	99	Death (1 year post-op due to pulmonary infection)
5	Male	5	2019-10-31	Yes	Left lobe without MHV	No splenectomy	No	75.8	Yes	130	Liver and kidney function normalized
6	Male	6	2019-10-22	Yes	Deceased donor	No splenectomy	No	34.4	Yes	131	Death (3 days post-op due to uncontrolled intra-abdominal hemorrhage, hemorrhagic shock)
7	Female	38	2019-11-06	Yes	Deceased donor	No splenectomy	Yes	281	Yes	700	Normal liver function. Underwent kidney transplant in August 2024; currently alive.
8	Male	29	2024-05-14	Yes	Right lobe without MHV	Partial splenectomy	Yes	324	Yes	518	Death (6 months post-op, December 2024, sudden death at home, cause unknown)

## Discussion

Caroli disease (CD) is a rare congenital disorder characterized by multifocal segmental dilation of the intrahepatic bile ducts, often associated with varying degrees of cystic kidney disease. It is distinguished by bile duct dilation without other significant liver abnormalities [4]. In contrast, Caroli syndrome (CS) involves bile duct dilation along with congenital hepatic fibrosis [5]. Caroli disease is extremely rare, with an estimated prevalence of approximately 1 in 1,000,000, while Caroli syndrome is more common, with a prevalence of around 1 in 100,000 [6]. CS is frequently associated with autosomal recessive polycystic kidney disease (ARPKD), which is caused by pathogenic mutations in the polycystic kidney and hepatic disease 1 (PKHD1) gene [1]. The PKHD1 gene is primarily expressed in the kidneys, with lower levels of expression in the liver, pancreas, and lungs. This expression pattern aligns with the disease phenotype, which primarily affects the liver and kidneys. Other studies have suggested that mutations in the WDR19 gene can also lead to either Caroli disease or Caroli syndrome [7].Due to limited resources, none of the cases in this cohort underwent PKHD1 or ARPKD genetic testing.

Clinically, Caroli syndrome (CS) presents with a wide range of symptoms, but commonly includes right upper quadrant pain, fever, jaundice, pruritus, and, in some cases, signs of portal hypertension. As the disease progresses, recurrent cholangitis, abscesses, sepsis, pancreatitis, and even cholangiocarcinoma may develop, complicating the clinical course [8]. Therefore, early diagnosis of Caroli disease (CD) and CS in patients with recurrent cholangitis is essential, as it enables the identification of the best treatment options, including liver resection and liver transplantation [9]. Research has shown that identifying the “central dot sign” within dilated bile ducts in the early stages of the disease can aid in the early diagnosis of CD [10–12]. This study, through a retrospective analysis of clinical data from 20 patients with Caroli syndrome who underwent liver transplantation, confirmed the effectiveness of liver transplantation in treating this disease and its long-term survival benefits. The 1-year, 3-year, and 5-year survival rates for the cohort were 90%, 85%, and 78.5%, respectively, which align with the reported survival rates for Caroli syndrome patients in the UNOS/OPTN database (approximately 90% 1-year survival) [13] and the findings of Habib et al. [14], further supporting liver transplantation as a curative treatment for end-stage Caroli syndrome.

In this study, 13 patients (65%) underwent living-donor liver transplantation (LDLT), and 7 patients (35%) received deceased-donor liver transplantation (DDLT). No significant difference in survival rates was observed between the two groups. This finding suggests that LDLT can be an effective alternative to DDLT in situations where donor availability is limited, particularly for pediatric patients, who comprised 75% of this cohort. The technical feasibility and safety of LDLT were confirmed through appropriate graft weight matching (224～1327g) and graft-to-recipient weight ratio (GRWR) (0.76%～4.90%). Additionally, 3 patients underwent simultaneous partial splenectomy, likely to address portal hypertension-related complications, although the long-term impact on prognosis requires further investigation. This relatively young age profile is primarily due to the characteristics of our center, which specializes in pediatric liver transplantation. As one of the major referral centers for pediatric liver transplant candidates in Northern China, a large proportion of Caroli syndrome patients treated at our institution are children, resulting in a younger cohort compared to previously published studies.

In terms of long-term postoperative complications, chronic renal failure (6 cases, 30%) and acute rejection (4 cases, 20%) were the most prominent. The onset of chronic renal failure (with a median onset time ranging from 12.1 to 46.7 months) was closely associated with preoperative comorbid polycystic kidney disease (16 cases, 80%) and postoperative nephrotoxicity from calcineurin inhibitors (CNI). Literature suggests that Caroli syndrome patients often have associated autosomal recessive polycystic kidney disease (ARPKD), and renal function may continue to deteriorate after liver transplantation. In this study, one patient eventually required a kidney transplant. Therefore, for patients with pre-existing renal impairment (2 cases) or polycystic kidney disease, it is recommended to reduce or substitute CNI drugs (e.g., by using sirolimus in combination with mycophenolate mofetil) to protect renal function. Alternatively, combined liver-kidney transplantation (CLKT) should be prioritized, especially for those requiring renal replacement therapy (RRT) [1].

The incidence of chronic renal failure before surgery was significantly higher in the adult group compared to the pediatric group (*p* = 0.010), likely due to the longer disease course and more severe renal involvement in adults. Additionally, postoperative AST levels were significantly higher in the pediatric group than in the adult group (*p* = 0.008), suggesting differences in liver metabolism and repair mechanisms between children and adults. However, the clinical significance of this finding requires further evaluation, particularly in conjunction with long-term follow-up. It is worth noting that, despite the pediatric group having more preoperative comorbidities (e.g., biliary tract infections, congenital hepatic fibrosis), their survival rate was not significantly different from that of the adult group. This underscores the feasibility of liver transplantation in pediatric patients.

Although no cases of cholangiocarcinoma were observed in this study, literature reports indicate that patients with Caroli disease/syndrome are at significantly higher risk for cholangiocarcinoma, with the risk increasing by up to 100 times compared to the general population [15, 16]. Therefore, long-term follow-up should include enhanced imaging surveillance (such as MRI/MRCP) and screening for tumor markers (e.g., CA19-9). Additionally, the occurrence of acute rejection (4 cases) and secondary rejection (3 cases) after surgery highlights the need for individualized adjustments to immunosuppressive regimens. For patients with recurrent rejection episodes, exploring precision medication strategies based on immune cell function testing could help strike a balance between managing rejection and minimizing the risks of infection or tumor development due to excessive immunosuppression.

In our cohort, the incidence of biopsy-proven acute rejection was 20% (4 out of 20 patients), which is comparable to the reported incidence of 25%～46% in the general liver transplant population [17]. Compared to recipients of liver transplants for other cholestatic conditions—such as primary sclerosing cholangitis (PSC) and primary biliary cholangitis (PBC)—the incidence of acute rejection in our cohort appears to be relatively low. Prior studies have demonstrated that PSC patients may have a higher rate of acute cellular rejection (ACR) following liver transplantation, ranging from 17 to 67%, especially in those with concomitant inflammatory bowel disease (IBD), which is thought to enhance alloimmune responses [18–20]. Additionally, chronic rejection and vanishing bile duct syndromehave been reported in 8%-13% of PSC recipients, significantly affecting both graft and patient survival. Similarly, PBC is recognized as an autoimmune-mediated cholestatic disease and has also been associated with a relatively high incidence of ACR post-transplant, with reported rates ranging from 21.7 to 83.3%. ACR in these patients not only negatively impacts recipient and graft survival but may also contribute to the recurrence of PBC (rPBC) after transplantation [21].The rejection rate observed in Caroli syndrome patients may be influenced by multiple factors, including underlying disease characteristics, immunological profile, and post-transplant management. First, Caroli disease is frequently associated with genetic mutations such as PKHD1, which are involved in ciliary dysfunction and may contribute to abnormal immune regulation. This underlying immunogenetic predisposition might enhance the risk of alloimmune responses. Second, most patients experience recurrent episodes of cholangitis prior to transplantation, often necessitating repeated antibiotic treatments and interventional biliary procedures. This persistent biliary inflammation may lead to chronic immune activation and antigen sensitization, predisposing patients to heightened immune reactivity post-transplant. Third, many patients with Caroli syndrome present with comorbidities such as renal dysfunction, systemic infections, or other ciliopathy-related conditions that may complicate immunosuppressive management. These factors may necessitate dose adjustments or modifications in immunosuppressive regimens, potentially leading to suboptimal immune control and increased rejection risk. There was no clear association between the type of immunosuppressive regimen and the occurrence of acute rejection. This suggests that rejection may be influenced more by individual immunologic factors, prior sensitization, or disease-specific immune mechanisms, rather than by the specific immunosuppressive protocol used. Although donor characteristics (e.g., donor age or type) could not be systematically assessed due to incomplete data, they may nonetheless contribute to the risk of rejection.

This study has several limitations. First, it is a single-center retrospective analysis with a relatively small sample size (*n* = 20), which may introduce selection bias and limit the generalizability of the findings. Second, the maximum follow-up period was 10 years, which may be insufficient to capture late-onset complications such as cholangiocarcinoma or ultra-long-term post-transplant outcomes.

Moreover, as with all retrospective studies, there is an inherent risk of data missingness and misclassification. Missing data may reduce the completeness and accuracy of the analysis, while misclassification may result from documentation errors or inconsistent coding practices. We made every effort to mitigate these issues by conducting thorough chart reviews and cross-verification of clinical records.Finally, the presence of unmeasured or residual confounding is an unavoidable limitation in retrospective observational studies. Despite our efforts to include key clinical variables, it is possible that unidentified confounders influenced some of the observed associations.

To overcome these limitations, we believe that future multi-center, large-scale prospective studies are warranted. Additionally, the inclusion of molecular and genetic biomarkers—such as PKHD1 mutation analysis—may help refine patient selection, optimize timing of transplantation, and improve individualized post-transplant management.

## Data Availability

The de-identified datasets generated and/or analyzed during the current study are available from the corresponding author upon reasonable request. Raw data are not publicly accessible to protect patient privacy.
